# A Survey of Deep Learning for Lung Disease Detection on Medical Images: State-of-the-Art, Taxonomy, Issues and Future Directions

**DOI:** 10.3390/jimaging6120131

**Published:** 2020-12-01

**Authors:** Stefanus Tao Hwa Kieu, Abdullah Bade, Mohd Hanafi Ahmad Hijazi, Hoshang Kolivand

**Affiliations:** 1 Faculty of Science and Natural Resources, Universiti Malaysia Sabah, Kota Kinabalu 88400, Sabah, Malaysia; stefanuskieu@gmail.com (S.T.H.K.); abb@ums.edu.my (A.B.); 2 Faculty of Computing and Informatics, Universiti Malaysia Sabah, Kota Kinabalu 88400, Sabah, Malaysia; 3 School of Computer Science and Mathematics, Liverpool John Moores University, Liverpool L3 3AF, UK; H.Kolivand@ljmu.ac.uk

**Keywords:** deep learning, lung disease detection, taxonomy, medical images

## Abstract

The recent developments of deep learning support the identification and classification of lung diseases in medical images. Hence, numerous work on the detection of lung disease using deep learning can be found in the literature. This paper presents a survey of deep learning for lung disease detection in medical images. There has only been one survey paper published in the last five years regarding deep learning directed at lung diseases detection. However, their survey is lacking in the presentation of taxonomy and analysis of the trend of recent work. The objectives of this paper are to present a taxonomy of the state-of-the-art deep learning based lung disease detection systems, visualise the trends of recent work on the domain and identify the remaining issues and potential future directions in this domain. Ninety-eight articles published from 2016 to 2020 were considered in this survey. The taxonomy consists of seven attributes that are common in the surveyed articles: image types, features, data augmentation, types of deep learning algorithms, transfer learning, the ensemble of classifiers and types of lung diseases. The presented taxonomy could be used by other researchers to plan their research contributions and activities. The potential future direction suggested could further improve the efficiency and increase the number of deep learning aided lung disease detection applications.

## 1. Introduction

Lung diseases, also known as respiratory diseases, are diseases of the airways and the other structures of the lungs [1]. Examples of lung disease are pneumonia, tuberculosis and Coronavirus Disease 2019 (COVID-19). According to Forum of International Respiratory Societies [2], about 334 million people suffer from asthma, and, each year, tuberculosis kills 1.4 million people, 1.6 million people die from lung cancer, while pneumonia also kills millions of people. The COVID-19 pandemic impacted the whole world [3], infecting millions of people and burdening healthcare systems [4]. It is clear that lung diseases are one of the leading causes of death and disability in this world. Early detection plays a key role in increasing the chances of recovery and improve long-term survival rates [5,6]. Traditionally, lung disease can be detected via skin test, blood test, sputum sample test [7], chest X-ray examination and computed tomography (CT) scan examination [8]. Recently, deep learning has shown great potential when applied on medical images for disease detection, including lung disease.

Deep learning is a subfield of machine learning relating to algorithms inspired by the function and structure of the brain. Recent developments in machine learning, particularly deep learning, support the identification, quantification and classification of patterns in medical images [9]. These developments were made possible due to the ability of deep learning to learned features merely from data, instead of hand-designed features based on domain-specific knowledge. Deep learning is quickly becoming state of the art, leading to improved performance in numerous medical applications. Consequently, these advancements assist clinicians in detecting and classifying certain medical conditions efficiently [10].

Numerous works on the detection of lung disease using deep learning can be found in the literature. To the best of our knowledge, however, only one survey paper has been published in the last five years to analyse the state-of-the-art work on this topic [11]. In that paper, the history of deep learning and its applications in pulmonary imaging are presented. Major applications of deep learning techniques on several lung diseases, namely pulmonary nodule diseases, pulmonary embolism, pneumonia, and interstitial lung disease, are also described. In addition, the analysis of several common deep learning network structures used in medical image processing is presented. However, their survey is lacking in the presentation of taxonomy and analysis of the trend of recent work. A taxonomy shows relationships between previous work and categorises them based on the identified attributes that could improve reader understanding of the topic. Analysis of trend, on the other hand, provides an overview of the research direction of the topic of interest identified from the previous work. In this paper, a taxonomy of deep learning applications on lung diseases and a trend analysis on the topic are presented. The remaining issues and possible future direction are also described.

The aims of this paper are as follows: (1) produce a taxonomy of the state-of-the-art deep learning based lung disease detection systems; (2) visualise the trends of recent work on the domain; and (3) identify the remaining issues and describes potential future directions in this domain. This paper is organised as follows. Section 2 presents the methodology of conducting this survey. Section 3 describes the general processes of using deep learning to detect lung disease in medical images. Section 4 presents the taxonomy, with detailed explanations of each subtopic within the taxonomy. The analysis of trend, research gap and future directions of lung disease detection using deep learning are presented in Section 5. Section 6 describes the limitation of the survey. Section 7 concludes this paper.

## 2. Methodology

**Figure 1:**
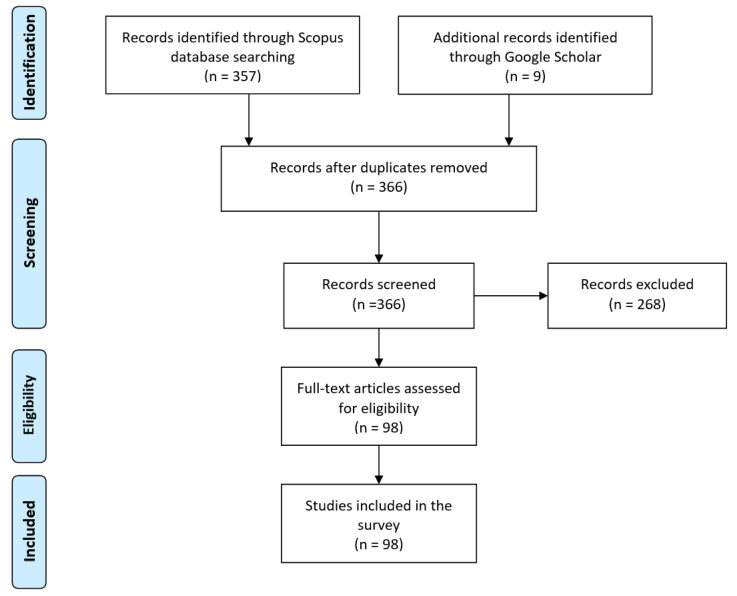
Flow diagram of the methodology used to conduct this survey.

In this section, the methodology used to conduct the survey of recent lung disease detection using deep learning is described. Figure 1 shows the flowchart of the methodology used.

First, a suitable database, as a main source of reference, of articles was identified. The Scopus database was selected as it is one of the largest databases of scientific peer-reviewed articles. However, several significant articles, indexed by Google Scholar but not Scopus, are also included based on the number of citations that they have received. Some preprint articles on COVID-19 are also included as the disease has just recently emerged. To ensure that this survey only covers the state-of-the-art works, only articles published recently (2016–2020) are considered. However, several older but significant articles are included too. To search for all possible deep learning aided lung disease detection articles, relevant keywords were used to search for the articles. The keywords used were “deep learning”, “detection”, “classification”, “CNN”, “lung disease”, “Tuberculosis”, “pneumonia”, “lung cancer”, “COVID-19” and “Coronavirus”. Studies were limited to articles written in English only. At the end of this phase, we identified 366 articles.

Second, to select only the relevant works, screening was performed. During the screening, only the title and abstract were assessed. The main selection criteria were this survey is only interested in work, whereby deep learning algorithms were applied to detect the relevant diseases. Articles considered not relevant were excluded. Based on the screening performed, only 98 articles were shortlisted.

Last, for all the articles screened, the eligibility inspection was conducted. Similar criteria, as in the screening phase, were used, whereby the full-text inspection of the articles was performed instead. All 98 screened articles passed this phase and were included in this survey. Out of the eligible articles, 90 were published in 2018 and onwards. This signifies that lung disease detection using deep learning is still a very active field. Figure 1 shows the numbers of studies identified, screened, assessed for eligibility and included in this survey.

## 3. The Basic Process to Apply Deep Learning for Lung Disease Detection

**Figure 2:**
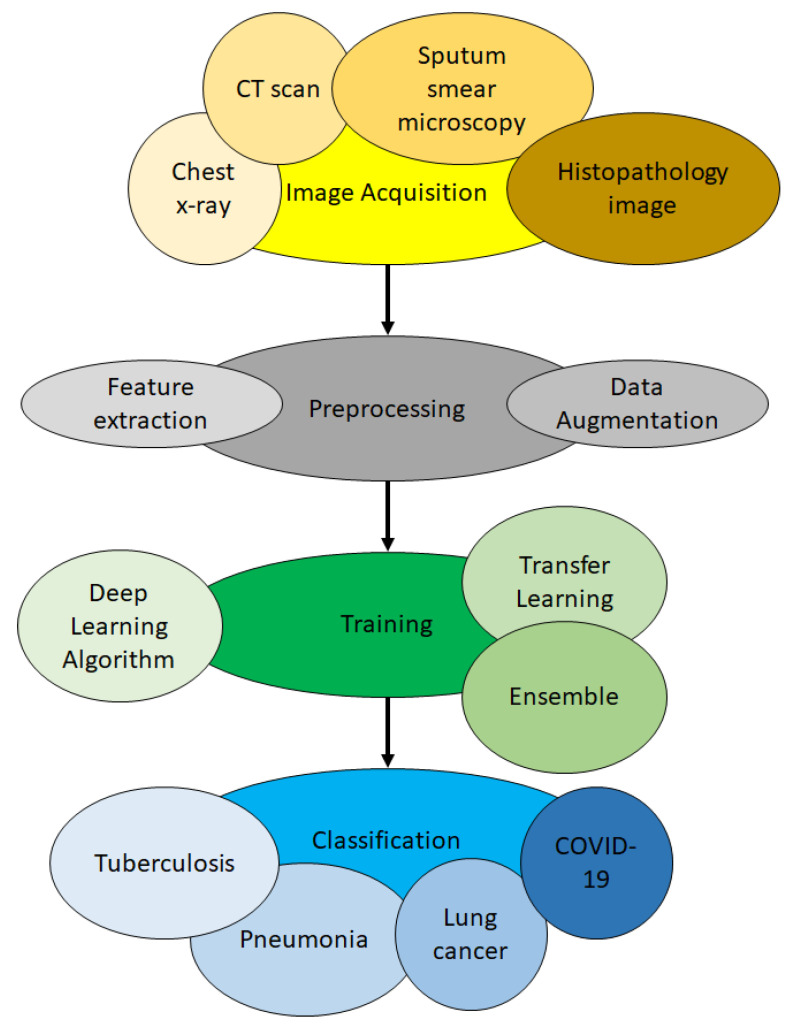
Overview of using deep learning for lung disease detection.

In this section, the process of how deep learning is applied to identify lung diseases from medical images is described. There are mainly three steps: image preprocessing, training and classification. Lung disease detection generally deals with classifying an image into healthy lungs or disease-infected lungs. The lung disease classifier, sometimes known as a model, is obtained via training. Training is the process in which a neural network learns to recognise a class of images. Using deep learning, it is possible to train a model that can classify images into their respective class labels. Therefore, to apply deep learning for lung disease detection, the first step is to gather images of lungs with the disease to be classified. The second step is to train the neural network until it is able to recognise the diseases. The final step is to classify new images. Here, new images unseen by the model before are shown to the model, and the model predicts the class of those images. The overview of the process is illustrated in Figure 2.

### 3.1. Image Acquisition Phase

The first step is to acquire images. To produce a classification model, the computer needs to learn by example. The computer needs to view many images to recognise an object. Other types of data, such as time series data and voice data, can also be used to train deep learning models. In the context of the work surveyed in this paper, the relevant data required to detect lung disease will be images. Images that could be used include chest X-ray, CT scan, sputum smear microscopy and histopathology image. The output of this step is images that will later be used to train the model.

### 3.2. Preprocessing Phase

The second step is preprocessing. Here, the image could be enhanced or modified to improve image quality. Contrast Limited Adaptive Histogram Equalisation (CLAHE) could be performed to increase the contrast of the images [12]. Image modification such as lung segmentation [13] and bone elimination [14] could be used to identify the region of interest (ROI), whereby the detection of the lung disease can then be performed on the ROI. Edge detection could also be used to provide an alternate data representation [15]. Data augmentation could be applied to the images to increase the amount of available data. Feature extraction could also be conducted so that the deep learning model could identify important features to identify a certain object or class. The output of this step is a set of images whereby the quality of the images is enhanced, or unwanted objects have been removed. The output of this step is images that were enhanced or modified that will later be used in training.

### 3.3. Training Phase

In the third step, namely training, three aspects could be considered. These aspects are the selection of deep learning algorithm, usage of transfer learning and usage of an ensemble. There are numerous deep learning algorithm, for example deep belief network (DBN), multilayer perceptron neural network (MPNN), recurrent neural network (RNN) and the aforementioned CNN. Different algorithms have different learning styles. Different types of data work better with certain algorithms. CNN works particularly well with images. Deep learning algorithm should be chosen based on the nature of the data at hand. Transfer learning refers to the transfer of knowledge from one model to another. Ensemble refers to the usage of more than one model during classification. Transfer learning and ensemble are techniques used to reduce training time, improve classification accuracy and reduce overfitting [16]. Further details concerning these two aspects could be found in Section 4.5 and Section 4.6, respectively. The output of this step is models generated from the data learned.

### 3.4. Classification Phase

In the fourth and final step, which is classification, the trained model will predict which class an image belongs to. For example, if a model was trained to differentiate X-ray images of healthy lungs and tuberculosis-infected lungs, it should be able to correctly classify new images (images that are never seen by the model before) into healthy lungs or tuberculosis-infected lungs. The model will give a probability score for the image. The probability score represents how likely an image belongs to a certain class. At the end of this step, the image will be classified based on the probability score given to it by the model.

## 4. The Taxonomy of State-Of-The-Art Work on Lung Disease Detection Using Deep Learning

**Figure 3:**
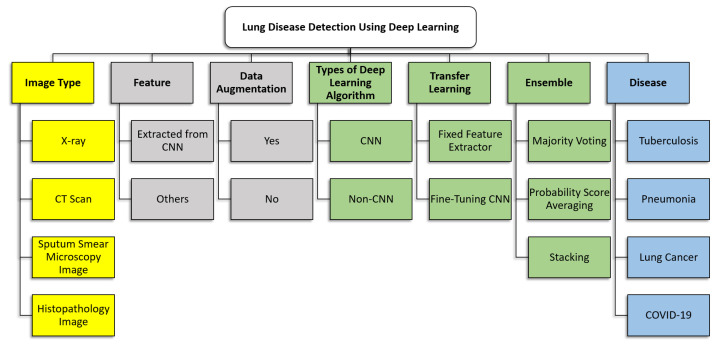
Taxonomy of lung disease detection using deep learning.

In this section, a taxonomy of the recent work on lung disease detection using deep learning is presented, which is the first contribution of this paper. The taxonomy is built to summarise and provide a clearer picture of the key concepts and focus of the existing work. Seven attributes were identified for inclusion in the taxonomy. These attributes were chosen as they were imminent and can be found in all the articles being surveyed. The seven attributes included in the taxonomy are image types, features, data augmentation, types of deep learning algorithms, transfer learning, the ensemble of classifiers and types of lung diseases. Section 4.1, Section 4.2, Section 4.3, Section 4.4, Section 4.5, Section 4.6 and Section 4.7 describe each attribute in detail, whereby the review of relevant works is provided. Section 4.8 describes the datasets used by the works surveyed. Figure 3 shows the taxonomy of state-of-the-art lung disease detection using deep learning.

### 4.1. Image Type

In the papers surveyed, four types of images were used to train the model: chest X-ray, CT scans, sputum smear microscopy images and histopathology images. These images are described in detail in Section 4.1.1, Section 4.1.2, Section 4.1.3 and Section 4.1.4. It should be noted that there are other imaging techniques exist such as positron emission tomography (PET) and magnetic resonance imaging (MRI) scans. Both PET and MRI scans could also be used to diagnose health conditions and evaluate the effectiveness of ongoing treatment. However, none of the papers surveyed used PET or MRI scans.

#### 4.1.1. Chest X-rays

**Figure 4:**
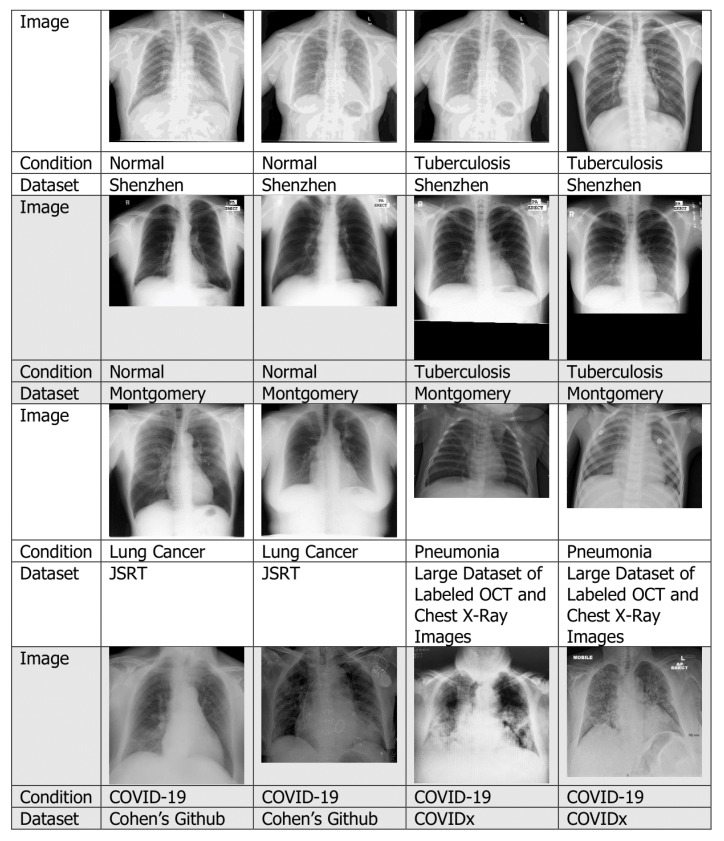
Examples of chest X-ray images.

An X-ray is a diagnostic test that helps clinicians identify and treat medical problems [17]. The most widely performed medical X-ray procedure is a chest X-ray, and a chest X-ray produces images of the blood vessels, lungs, airways, heart and spine and chest bones. Traditionally, medical X-ray images were exposed to photographic films, which require processing before they can be viewed. To overcome this problem, digital X-rays are used [18]. Figure 4 shows several examples of chest X-ray with different lung conditions taken from various datasets.

Among the papers surveyed, the majority of them used chest X-rays. For example, X-rays were used for tuberculosis detection [19], pneumonia detection [20], lung cancer detection [14] and COVID-19 detection [21].

#### 4.1.2. CT Scans

**Figure 5:**
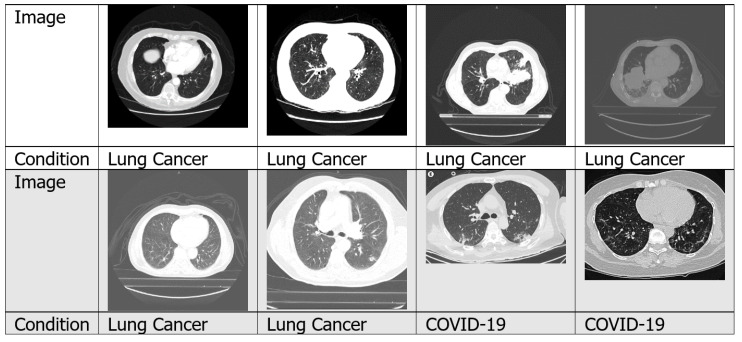
Examples of CT scan images.

A CT scan is a form of radiography that uses computer processing to create sectional images at various planes of depth from images taken around the patient’s body from different angles [22]. The image slices can be shown individually, or they can be stacked to produce a 3D image of the patient, showing the tissues, organs, skeleton and any abnormalities present [23]. CT scan images deliver more detailed information than X-rays. Figure 5 shows examples of CT scan images taken from numerous datasets. CT scans have been used to detect lung disease in numerous work found in the literature, for example for tuberculosis detection [24], lung cancer detection [25] and COVID-19 detection [26].

#### 4.1.3. Sputum Smear Microscopy Images

**Figure 6:**
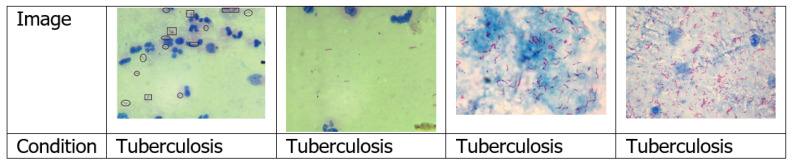
Examples of sputum smear microscopy images.

Sputum is a dense fluid formed in the lungs and airways leading to the lungs. To perform sputum smear examination, a very thin layer of the sputum sample is positioned on a glass slide [27]. Among the papers surveyed, only five used sputum smear microscopy image [28,29,30,31,32]. Figure 6 shows examples of sputum smear microscopy images.

#### 4.1.4. Histopathology Images

**Figure 7:**
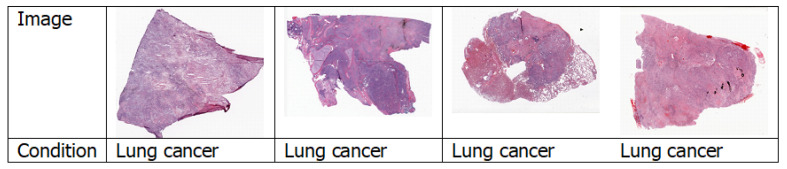
Examples of histopathology images.

Histopathology is the study of the symptoms of a disease through microscopic examination of a biopsy or surgical specimen using glass slides. The sections are dyed with one or more stains to visualise the different components of the tissue [33]. Figure 7 shows a few examples of histopathology images. Among all the papers surveyed, only Coudray et al. [34] used histopathology images.

### 4.2. Features

In computer vision, features are significant information extracted from images in terms of numerical values that could be used to solve specific problem [35]. Features might be in the form of specific structures in the image such as points, edges, colour, sizes, shapes or objects. Logically, the types of images affect the quality of the features.

Feature transformation is a process that creates new features using the existing features. These new features may not have the same representation as to the original features, but they may have more discriminatory power in a different space than the original space. The purpose of feature transformation is to provide a more useful feature for the machine learning algorithm for object identification. The features used in the surveyed papers include: Gabor, GIST, Local binary patterns (LBP), Tamura texture descriptor, colour and edge direction descriptor (CEDD) [36], Hu moments, colour layout descriptor (CLD) edge histogram descriptor (EHD) [37], primitive length, edge frequency, autocorrelation, shape features, size, orientation, bounding box, eccentricity, extent, centroid, scale-invariant feature transform (SIFT), regional properties area and speeded up robust features (SURF) [38]. Other feature representations in terms of histograms include pyramid histogram of oriented gradients (PHOG), histogram of oriented gradients (HOG) [39], intensity histograms (IH), shape descriptor histograms (SD), gradient magnitude histograms (GM), curvature descriptor histograms (CD) and fuzzy colour and texture histogram (FCTH). Some studies even performed lung segmentations before training their models (e.g., [13,14,36]).

From the literature, a majority of the works surveyed used features that are automatically extracted from CNN. CNN can automatically learn and extract features, discarding the need for manual feature generation [40].

### 4.3. Data Augmentation

In deep learning, it is very important to have a large training dataset, as the community agrees that having more images can help improve training accuracy. Even a weak algorithm with a large amount of data can be more accurate than a strong algorithm with a modest amount of data [41]. Another obstacle is imbalanced classes. When doing binary classification training, if the number of samples of one class is a lot higher than the other class, the resulting model would be biased [6]. Deep learning algorithms perform optimally when the amount of samples in each class is equal or balanced.

**Figure 8:**
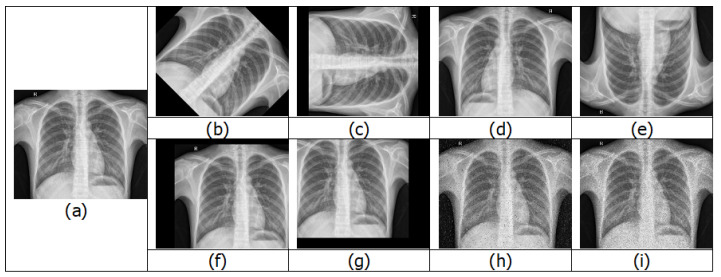
Examples of image augmentation: (**a**) original; (**b**) 45° rotation; (**c**) 90° rotation; (**d**) horizontal flip; (**e**) vertical flip; (**f**) positive x and y translation; (**g**) negative x and y translation; (**h**) salt and pepper noise; and (**i**) speckle noise.

One way to increase the training dataset without obtaining new images is to use image augmentation. Image augmentation creates variations of the original images. This is achieved by performing different methods of processing, such as rotations, flips, translations, zooms and adding noise [42]. Figure 8 shows various examples of images after image augmentation.

Data augmentation can also help increase the amount of relevant data in the dataset. For example, consider a car dataset with two labels, X and Y. One subset of the dataset contains images of cars of label X, but all the cars are facing left. The other subset contains images of cars of label Y, but all the cars are facing right. After training, a test image of a label Y car facing left is fed into the model, and the model labels that the car as X. The prediction is wrong as the neural network search for the most obvious features that distinguish one class from another. To prevent this, a simple solution is to flip the images in the existing dataset horizontally such that they face the other side. Through augmentation, we may introduce relevant features and patterns, essentially boosting overall performance.

Data augmentation also helps prevent overfitting. Overfitting refers to a case where a network learns a very high variance function, such as the perfect modelling of training results. Data augmentation addresses the issue of overfitting by introducing the model with more diverse data [43]. This diversity in data reduces variance and improves the generalisation of the model.

However, data augmentation cannot overcome all biases present in a small dataset [43]. Other disadvantages of data augmentation include additional training time, transformation computing costs and additional memory costs.

### 4.4. Types of Deep Learning Algorithm

**Figure 9:**
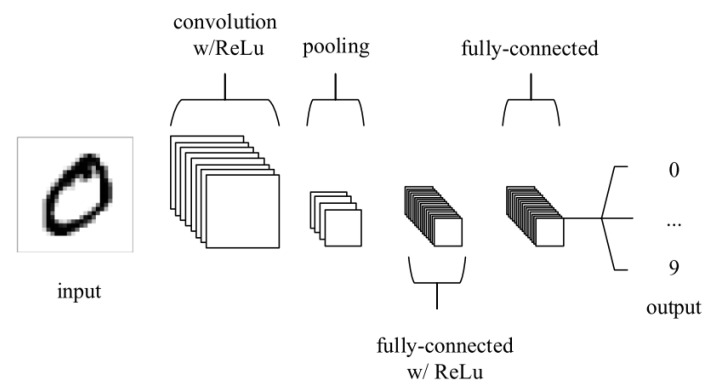
Example of a CNN structure.

The most common deep learning algorithm, CNN, is especially useful to find patterns in images. Similar to the neural networks of the human brain, CNNs consist of neurons with trainable biases and weights. Each neuron receives several inputs. Then, a weighted sum over the inputs is computed. The weighted sum is then passed to an activation function, and an output is produced. The difference between CNN and other neural networks is that CNN has convolution layers. Figure 9 shows an example of a CNN architecture [44]. A CNN consists of multiple layers, and the four main types of layers are convolutional layer, pooling layer and fully-connected layer. The convolutional layer performs an operation called a “convolution”. Convolution is a linear operation involving the multiplication of a set of weights with the input. The set of weights is called a kernel or a filter. The input data are larger than the filter. The multiplication between a filter-sized section of the input and the filter is a dot product. The dot product is then summed, resulting in a single value. The pooling layer gradually reduces the spatial size of the representation to lessen the number of parameters and computations in the network, thus controlling overfitting. A rectified linear unit (ReLu) is added to the CNN to apply an elementwise activation function such as sigmoid to the output of the activation produced by the previous layer. More details of CNN can be found in [44,45].

CNN generally has two components when learning, which are feature extraction and classification. In the feature extraction stage, convolution is implemented on the input data using a filter or kernel. Then, a feature map is subsequently generated. In the classification stage, the CNN computes a probability of the image belongs to a particular class or label. CNN is especially useful for image classification and recognition as it automatically learns features without needing manual feature extraction [40]. CNN also can be retrained and applied to a different domain using transfer learning [46]. Transfer learning has been shown to produce better classification results [19].

Another deep learning algorithm is DBN. DBN can be defined as a stack of restricted Boltzmann machines (RBM) [47]. The layer of the DBN has two functions, except for the first and final layers. The layer serves as the hidden layer for the nodes that come before it, and as the input layer for the nodes that come after it. The first RBM is designed to reproduce as accurately as possible the input to train a DBN. Then, the hidden layer of the first RBM is treated as the visible layer for the second one, and the second RBM is trained using the outputs from the first RBM. This process keeps repeating until every layer of the network is trained. After this initial training, the DBN has created a model that can detect patterns in the data. DBN can be used to recognise objects in images, video sequences and motion-capture data. More details of DBN can be found in [31,48].

One more example of a deep learning algorithm used in the papers surveyed is a bag of words (BOW) model. BOW is a method to extract features from the text for use in modelling. In BOW, the number of the appearance of each word in a document is counted, then the frequency of each word was examined to identify the keywords of the document, and a frequency histogram is made. This concept is similar to the bag of visual words (BOVW), sometimes referred to as bag-of-features. In BOVW, image features are considered as the “words”. Image features are unique patterns that were found in an image. The general idea of BOVW is to represent an image as a set of features, where each feature contains keypoints and descriptors. Keypoints are the most noticeable points in an image, such that, even if the image is rotated, shrunk or enlarged, its keypoints are always the same. A descriptor is the description of the keypoint. Keypoints and descriptors are used to construct vocabularies and represent each image as a frequency histogram of features. From the frequency histogram, one can find other similar images or predict the class of the image. Lopes and Valiati proposed Bag of CNN features to classify tuberculosis [19].

### 4.5. Transfer Learning

Transfer learning emerged as a popular method in computer vision because it allows accurate models to be built [49]. With transfer learning, a model learned from a domain can be re-used on a different domain. Transfer learning can be performed with or without a pre-trained model.

A pre-trained model is a model developed to solve a similar task. Instead of creating a model from scratch to solve a similar task, the model trained on other problem is used as a starting point. Even though a pre-trained model is trained on a task which is different from the current task, the features learned, in most cases, found to be useful for the new task. The objective of training a deep learning model is to find the correct weights for the network by numerous forward and backward iterations. By using pre-trained models that have been previously trained on large datasets, the weights and architecture obtained can be used and applied to the current problem. One of the advantages of a pre-trained model is the reduced cost of training for the new model [50]. This is because pre-trained weights were used, and the model only has to learn the weights of the last few layers.

Many CNN architectures are pre-trained on ImageNet [51]. The images were gathered from the internet and labelled by human labellers using Amazon’s Mechanical Turk crowd-sourcing tool. ILSVRC uses a subset of ImageNet with approximately 1000 images in each of 1000 classes. Altogether, there are approximately 1.2 million training images, 50,000 validation images and 150,000 testing images.

Transfer learning can be used in two ways: (i) fine-tuning; or (ii) using CNN as a feature extractor. In fine-tuning, the weights of the pre-trained CNN model are preserved on some of the layers and tuned in the others [52]. Usually, the weights of the initial layers of the model are frozen while only the higher layers are retrained. This is because the features obtained from the first layers are generic (e.g., edge detectors or colour blob detectors) and applicable to other tasks. The top-level layers of the pre-trained models are retrained so that the model learned high-level features specific to the new dataset. This method is typically recommended if the training dataset is huge and very identical to the original dataset that the pre-trained model was trained on. On the other hand, CNN is used as a feature extractor. This is conducted by removing the last fully-connected layer (the one which outputs the probabilities for being in each of the 1000 classes from ImageNet) and then using the network as a fixed feature extractor for the new dataset [53]. For tasks where only a small dataset is available, it is usually recommended to take advantage of features learned by a model trained on a larger dataset in the same domain. Then, a classifier is trained from the features extracted.

There are several issues that need to be considered when using transfer learning: (i) ensuring that the pre-trained model selected has been trained on a similar dataset as the new target dataset; and (ii) using a lower learning rate for CNN weights that are being fine-tuned, because the CNN weights are expected to be relatively good, and we do not wish to distort them too quickly and too much [53].

### 4.6. Ensemble of Classifiers

When more than one classifier is combined to make a prediction, this is known as ensemble classification [16]. Ensemble decreases the variance of predictions, therefore making predictions that are more accurate than any individual model. From work found in the literature, the ensemble techniques used include majority voting, probability score averaging and stacking.

In majority voting, every model makes a prediction for each test instance, or, in other words, votes for a class label, and the final prediction is the label that received the most votes [54]. An alternate version of majority voting is weighted majority voting, in which the votes of certain models are deemed more important than others. For example, majority voting was used by Chouhan et al. [55].

In probability score averaging, the prediction scores of each model are added up and divided by the number of models involved [56]. An alternate version of this is weighted averaging, where the prediction score of each model is multiplied by the weight, and then their average is calculated. Examples of works which used probability score averaging are found in [15,57].

In stacking ensemble, an algorithm receives the outputs of weaker models as input and tries to learn how to best combine the input predictions to provide a better output prediction [58]. For example, stacking ensemble was used by Rajaraman et al. [12].

### 4.7. Type of Disease

In this section, the deep learning techniques applied for detecting tuberculosis, pneumonia, lung cancer and COVID-19 are discussed in greater detail in Section 4.7.1, Section 4.7.2, Section 4.7.3 and Section 4.7.4, respectively. The first three diseases were considered as they are the most common causes of critical illness and death worldwide related to lung [2], while COVID-19 is an ongoing pandemic [3]. We also found that most of the existing work was directed at detecting these specific lung-related diseases.

#### 4.7.1. Tuberculosis

Tuberculosis is a disease caused by Mycobacterium tuberculosis bacteria. According to the World Health Organisation, tuberculosis is among the ten most common causes of death in the world [59]. Tuberculosis infected 10 million people and killed 1.6 million in 2017. Early detection of tuberculosis is essential to increase the chances of recovery [5].

Two studies used Computer-Aided Detection for Tuberculosis (CAD4TB) for tuberculosis detection [60,61]. CAD4TB is a tool developed by Delft Imaging Systems in cooperation with the Radboud University Nijmegen and the Lung Institute in Cape Town. CAD4TB works by obtaining the patient’s chest X-ray, analysing the image via CAD4TB cloud server or CAD4TB box computer, generating a heat map of the patient’s lung and displaying an abnormality score from 0 to 100. Murphy et al. [60] showed that CAD4TB v6 is an accurate system, reaching the level of expert human readers. A technique for automated tuberculosis screening by combining X-ray-based computer-aided detection (CAD) and clinical information was introduced by Melendez et al. [61]. They combined automatic chest X-ray scoring by CAD with clinical information. This combination improved accuracies and specificities compared to the use of either type of information alone.

In the literature, several works use CNN to classify tuberculosis. A method that incorporated demographic information, such as age, gender and weight, to improve CNN’s performance was presented by Heo et al. [62]. Results indicate that CNN, including the demographic variables, has a higher area under the receiver operating characteristic curve (AUC) score and greater sensitivity then CNN based on chest X-rays images only. A simple convolutional neural network developed for tuberculosis detection was proposed by Pasa et al. [63]. The proposed approach is found to be more efficient than previous models but retains their accuracy. This method significantly reduced the memory and computational requirement, without sacrificing the classification performance. Another CNN-based model has been presented to classify different categories of tuberculosis [64]. A CNN model is trained on the region-based global and local features to generate new features. A support vector machine (SVM) classifier was then applied for tuberculosis manifestations recognition. CNN has also been used to classify tuberculosis [65,66,67]. Ul Abideen et al. [68] used a Bayesian-based CNN that exploits the model uncertainty and Bayesian confidence to improve the accuracy of tuberculosis identification. In other work, a deep CNN algorithm named deep learning-based automatic detection (DLAD), was developed for tuberculosis classification that contains 27 layers with 12 residual connections [69]. DLAD shows outstanding performance in tuberculosis detection when applied on chest X-rays, obtaining results better than physicians and thoracic radiologists.

Lopes and Valiati proposed Bag of CNN features to classify tuberculosis [19] where feature extraction is performed by ResNet, VggNet and GoogLenet. Then, each chest X-ray is separated into subregions whose size is equal to the input layer of the networks. Each subregion is regarded as a “feature”, while each X-ray is a “bag”.

Several works that utilised transfer learning are described in this paragraph. Hwang et al. obtained an accuracy of 90.3% and AUC of 0.964 using transfer learning from ImageNet and training on a dataset of 10848 chest X-rays [70]. Pre-trained GoogLeNet and AlexNet were used to perform pulmonary tuberculosis classification by Lakhani and Sundaram [57], who concluded that higher accuracy was achieved when using the pre-trained model. Their pre-trained AlexNet achieved an AUC of 0.98 and their pre-trained GoogLeNet achieved an AUC of 0.97. Lopes and Valiati used pre-trained GoogLenet, ResNet and VggNet architectures as features extractors and the SVM classifier to classify tuberculosis [19]. They achieved AUC of 0.900–0.912. Fine-tuned ResNet-50, ResNet-101, ResNet-512, VGG16, VGG19 and AlexNet were used by Islam et al. to classify tuberculosis. These models achieved an AUC of 0.85–0.91 [71]. Instead of using networks pre-trained from ImageNet, pre-training can be performed on other datasets, such as the NIH-14 dataset [72]. This dataset contains an assortment of diseases (which does not include tuberculosis) and is from the same modality as that of the data under consideration for tuberculosis. Experiments show that the features learned from the NIH dataset are useful for identifying tuberculosis. A study performed data augmentation and then compared the performances of three different pre-trained models to classify tuberculosis [73]. The results show that suitable data augmentation methods were able to rise the accuracies of CNNs. Transfer learning was also used by Abbas and Abdelsamea [74], Karnkawinpong and Limpiyakorn [75] and Liu et al. [76]. A coarse-to-fine transfer learning was applied by Yadav et al. [77]. First, the datasets are split according to the resolution and quality of the images. Then, transfer learning is applied to the low-resolution dataset first, followed by the high-resolution dataset. In this case, the model was first trained on the low-resolution NIH dataset, and then trained on the high-resolution Shenzen and Montgomery datasets. Sahlol et al. [78] used CNN as fixed feature extractor and Artificial Ecosystem-Based Optimisation to select the optimal subset of relevant features. KNN was used as the classifier.

Several works that utilised ensemble are described in this paragraph. An ensemble method using the weighted averages of the probability scores for the AlexNet and GoogLeNet algorithms was used by Lakhani and Sundaram [57]. In [79], ensemble by weighted averages of probability scores is used. An ensemble of six CNNs was developed by Islam et al. [71]. The ensemble models were generated by calculating the simple averaging of the probability predictions given by every single model. Another ensemble classifier was created by combining the classifier from the Simple CNN Feature Extraction and a classifier from Bag of CNN features proposals [19]. Three classifiers were trained, using the features from ResNet, GoogLenet and VggNet, respectively. The Simple Features Ensemble combines all three classifiers, and the output is obtained through a simple soft-voting scheme. A stacking ensemble for tuberculosis detection was proposed by Rajaraman et al. [12]. An ensemble generated via a feature-level fusion of neural network models was also used to classify tuberculosis [80]. Three models were employed: the DenseNet, ResNet and Inception-ResNet. As such, the ensemble was called RID network. Features were extracted using the RID network, and SVM was used as a classifier. Tuberculosis classification was also executed using another ensemble of three regular architectures: ResNet, AlexNet and GoogleNet [79]. Each architecture was trained from scratch, and different optimal hyper-parameter values were used. The sensitivity, specificity and accuracy of the ensemble were higher than when each of the regular architecture was used independently. The authors of [15,81] performed a probability score averaging ensemble of CNNs trained on features extracted from a different type of images; the enhanced chest X-ray images and the edge detected images of the chest X-ray. Rajaraman and Antani [82] studied and compared various ensemble methods that include majority voting and stacking. Results show that stacking ensemble achieved the highest classification accuracy.

Other techniques used to classify tuberculosis images include k-Nearest Neighbour (kNN), sequential minimal optimisation and simple linear regression [38]. A Multiple-Instance Learning-based approach was also attempted [83]. The advantage of this method is the lower labelling detail required during optimisation. In addition, the minimal supervision required allows easy retraining of a previously optimised system. One tuberculosis detection system uses ViDi Systems for image analysis of chest X-rays [84]. ViDi is an industrial-grade deep learning image analysis software developed by COGNEX. ViDi has shown feasible performance in the detection of tuberculosis. The authors of [36] introduced a fully automatic frontal chest screening system that is capable of detecting tuberculosis-infected lungs. This method begins with the segmentation of the lung. Then, features are extracted from the segmented images. Examples of features include shape and curvature histograms. Finally, a classifier was used to detect the disease.

For CT scans related tuberculosis detection works, a method called AECNN was proposed [85]. An AE-CNN block was formed by combining the feature extraction of CNN and the unsupervised features of AutoEncoder. The model then analyses the region of interest within the image to perform the classification of tuberculosis. A research study explores the use of CT pulmonary images to diagnose and classify tuberculosis at five levels of severity to track treatment effectiveness [24]. The tuberculosis abnormalities only occupy limited regions in the CT image, and the dataset is quite small. Therefore, depth-ResNet was proposed. Depth-ResNet is a 3D block-based ResNet combined with the injection of depth information at each layer. As an attempt to automate tuberculosis related lung deformities without sacrificing accuracy, advanced AI algorithms were studied to draw clinically actionable hypotheses [86]. This approach involves thorough image processing, subsequently performing feature extraction using TensorFlow and 3D CNN to further augment the metadata with the features extracted from the image data, and finally perform six class binary classification using the random forest. Another attempt for this problem was proposed by Zunair et al. [87]. They proposed a 16-layer 3D convolutional neural network with a slice selection. The goal is to estimate the tuberculosis severity based on the CT image. An integrated method based on optical flow and a characterisation method called Activity Description Vector (ADV) was presented to take care of the classification of chest CT scan images affected by different types of tuberculosis [88]. The important point of this technique is the interpretation of the set of cross-sectional chest images produced by CT scan, not as a volume but as a series of video images. This technique can extract movement descriptors capable of classifying tuberculosis affections by analysing deformations or movements generated in these video series. The idea of optical flow refers to the approximation of displacements of intensity patterns. In short, the ADV vector describes the activity in image series by counting for each region of the image the movements made in four directions of the 2D space.

For sputum microscopy images-related tuberculosis detection works, CNN was used for the detection and localisation of drug-sensitive tuberculosis bacilli in sputum microscopy images [29]. This method automatically localises bacilli in each view-field (a patch of the whole slide). A study found that, when training a CNN on three different image versions, namely RGB, R-G and grayscale, the best performance was achieved when using R-G images [28]. Image binarisation can also be used for preprocessing before the data were fed into a CNN [30]. Image binarisation is a segmentation method to classify the foreground and background of the microscopic sputum smear images. The segmented foreground consists of single bacilli, touching bacillus and other artefacts. A trained CNN is then given the foreground objects, and the CNN will classify the objects into bacilli and non-bacilli. Another tuberculosis detection system automatically attains all view-fields using a motorised microscopic stage [32]. After that, the data are delivered to the recognition system. A customised Inception V3 DeepNet model is used to learn from the pre-trained weights of Inception V3. Afterwards, the data were classified using SVM. DBN was also used to detect tuberculosis bacillus present in the stained microscopic images of sputum [31]. For segmentation, the Channel Area Thresholding algorithm is used. Location-oriented histogram and speed up robust feature (SURF) algorithm were used to extract the intensity-based local bacilli features. DBN is then used to classify the bacilli objects. Table 1 shows the summary of papers for tuberculosis detection using deep learning.

#### 4.7.2. Pneumonia

Pneumonia is a lung infection that causes pus and fluid to fill the alveoli in one or both lungs, thus making breathing difficult [89]. Symptoms include severe shortness of breath, chest pain, chills, cough, fever or fatigue. Community-acquired pneumonia is still a recurrent cause of morbidity and mortality [90]. Most of the studies used transfer learning and data augmentation. Tobias et al. [91] straightforwardly used CNN. Stephen et al. [92] trained their CNN from scratch while using rescale, rotation, width shift, height shift, shear, zoom and horizontal flip as their augmentation techniques. A pre-trained CNN was utilised by the authors of [20,55,93,94,95,96,97] for pneumonia detection, while the latter four also applied data augmentation on their training datasets. For data augmentation, random horizontal flipping was used by Rajpurkar et al. [96]; shifting, zooming, flipping and 40-degree angles rotation were used by Ayan and Ünver [20]; Chouhan et al. [55] used noise addition, random horizontal flip random resized crop and images intensity adjustment; and Rahman et al. [97] used rotation, scaling and translation. Hashmi et al. [98] used CNN with transfer learning, data augmentation and ensemble by weighted averaging.

In a unique study, Acharya and Satapathy [99] used Deep Siamese CNN architecture. Deep Siamese network uses the symmetric structure of the two input image for classification. Thus, the X-ray images were separated into two parts, namely the left half and the right half. Each half was then fed into the network to compare the symmetric structure together with the amount of the infection that is spread across these two regions. Training the model for both left and right parts of the X-ray images makes the classification process more robust. Elshennawy and Ibrahim [100] used CNN and Long Short-Term Memory (LSTM)-CNN for pneumonia detection. The key advantage of the LSTM is that it can model both long and short-term memory and can deal with the vanishing gradient problem by training on long strings and storing them in memory. Emhamed et al. [101] studied and compared seven different deep learning algorithms: Decision Tree, Random Forest, KNN, AdaBoost, Gradient Boost, XGBboost and CNN. Their results show CNN obtained the highest accuracy for pneumonia classification, followed by Random forest and XGBboost. Hashmi et al. [98] used CNN with transfer learning, data augmentation and ensemble by weighted averaging.

In addition, Kumar et al. [102] attempted not only pneumonia classification, but also ROI identification. Pneumonia was detected by looking at lung opacity, and Mask-RCNN based model was used to identify lung opacity that is likely to depict pneumonia. They also performed ensemble by combining confidence scores and bounding boxes. In addition to pneumonia detection, Hurt et al. [103] proposed an approach that provides a probabilistic map on the chest X-ray images to assist in the diagnosis of pneumonia. Table 2 shows the summary of papers for pneumonia detection using deep learning.

#### 4.7.3. Lung Cancer

One key characteristic of lung cancer is the presence of pulmonary nodules, solid clumps of tissue that appear in and around the lungs [104]. These nodules can be seen in CT scan images and can be malignant (cancerous) in nature or benign (not cancerous) [23].

As early as 2015, Hua et al. [105] used models of DBN and CNN to perform nodule classification in CT scans. They showed that, using deep learning, it is possible to seamlessly extract features for lung nodules classification into malignant or benign without computing the morphology and texture features. Rao et al. [25] and Kurniawan et al. [106] used CNN in a straightforward way to detect lung cancer in CT scans. Song et al. [23] compared the classification performance of CNN, deep neural network and stacked autoencoder (a multilayer sparse autoencoder of a neural network) and concluded that CNN has the highest accuracy among them. Ciompi et al. [107] used multi-stream multi-scale CNNs to classify lung nodules into six different classes: solid, non-solid, part-solid, calcified, perifissural and spiculated nodules. Specifically, they presented a multi-stream multi-scale architecture, in which CNN concurrently handles multiple triplets of 2D views of a nodule at multiple scales and then calculates the probability for the nodule in each of the six classes. Yu et al. [14] performed bone elimination and lung segmentation before training with CNN. Shakeel et al. [108] performed image denoising and enhanced the quality of the images, and then segmented the lungs by using the improved profuse clustering technique. Afterwards, a neural network is trained to detect lung cancer. The approach of Ardila et al. [13] consists of four components: lung segmentation, cancer region of interest detection model, full-volume model and cancer risk prediction model. After lung segmentation, the region of interest detection model proposes the most nodule-like regions, while the full-volume model was trained to predict cancer probability. The outputs of these two models were considered to generates the final prediction. Chen et al. [109] performed nodule enhancement and nodule segmentation before performing nodule detection.

For the works that employed transfer learning, Hosny et al. [110] and Xu et al. [111] both used CNN with data augmentation. For augmentations, both studies used flipping, translation and rotation. The authors of [112] leveraged the LUNA16 dataset to train a nodule detector and then refined that detector with the KDSB17 dataset to provide global features. Combining that and local features from a separate nodule classifier, they were able to detect lung cancer with high accuracy. The authors of [113] used transfer learning by training the model multiple times. It commenced using the more general images from the ImageNet dataset, followed by detecting nodules from chest X-rays in the ChestX-ray14 dataset, and finally detecting lung cancer nodules from the JSRT dataset. The authors of [34] is the only study surveyed to do lung cancer detection on histopathology images. Adenocarcinoma (LUAD) and squamous cell carcinoma (LUSC) are the most frequent subtypes of lung cancer, and visual examination by an experienced pathologist is needed to differentiate them. In this work, CNN was trained on histopathology slides images to automatically and accurately classify them into LUAD, LUSC or normal lung tissue. Xu et al. [114] used a CNN-long short-term memory network (LSTM) to detect lesions on chest X-ray images. Long short-term memory is an extension of RNN. This CNN-LSTM network offers probable clinical relationships between lesions to assist the model to attain better predictions. Table 3 shows the summary of papers for lung cancer detection using deep learning.

#### 4.7.4. COVID-19

COVID-19 is an infectious disease caused by a recently discovered coronavirus [115]. Senior citizens are those at high risk to develop severe sickness, along with those that have historical medical conditions such as cardiovascular disease, chronic respiratory disease, cancer and diabetes [116].

A straightforward approach to detect COVID-19 using CNN with transfer learning and data augmentation was used by Salman et al. [21]. For transfer learning, they used InceptionV3 as a fixed feature extractor. Other works that implemented the similar approach of transfer learning for COVID-19 detection can be found in [117,118,119,120,121,122].

The authors of [123,124] performed 3-class classification using CNN with transfer learning, classifying X-ray images into normal, COVID-19 and viral pneumonia cases. Chowdhury et al. [125] utilised CNN with transfer learning and data augmentation to classify classifying X-ray images into normal, COVID-19 and viral pneumonia cases. The augmentation techniques used were rotation, scaling and translation. Wang et al. [126] trained a CNN from scratch and data augmentation to perform three-class classification. The augmentation technique used were translation, rotation, horizontal flip and intensity shift. Other work performing three-class classification can be found in [4,127,128,129,130]. Studies that employ data augmentation to increase the amount of data available can be found in [131,132]. In addition to COVID-19 detection on X-ray images, Alazab et al. [131] managed to perform prediction on the number of COVID-19 confirmations, recoveries and deaths in Jordan and Australia.

For works utilising ensemble, Ouyang et al. [133] implemented weighted averaging ensemble. Mahmud et al. [134] implemented stacking ensemble, whereby the images were classified into four categoriesL normal, COVID-19, viral pneumonia and bacterial pneumonia.

Shi et al. [135] utilised VB-Net for image segmentation and feature extraction and used a modified random decision forests method for classification. Several handcrafted features were also calculated and used to train the random forest model. More information about random forest can be found in [136].

A system that receives thoracic CT images and points out suspected COVID-19 cases was proposed by Gozes et al. [26]. The system analyses CT images at two distinct subsystems. Subsystem A performed the 3D analysis of the case volume for nodules and focal opacities, while Subsystem B performed the 2D analysis of each slice of the case to detect and localise larger-sized diffuse opacities. In Subsystem A, nodules and small opacities detection were conducted using a commercial software. Besides the detection of abnormalities, the software also provided measurements and localisation. For Subsystem B, lung segmentation was first performed, and then COVID-19 related abnormalities detection was conducted using CNN with transfer learning and data augmentation. If an image is classified as positive, a localisation map was generated using the Grad-cam technique. To provide a complete review of the case, Subsystems A and B were combined. The final outputs include per slice localisation of opacities (2D), 3D volumetric presentations of the opacities throughout the lungs and a Corona score, which is a volumetric measurement of the opacities burden.

The authors of [137] focused on location-attention classification mechanism. First, the CT images were preprocessed. Second, a 3D CNN model was employed to segment several candidate image patches. Third, an image classification model was trained and employed to categorise all image patches into one of three classes: COVID-19, Influenza-A-viral-pneumonia and irrelevant-to-infection. A location-attention mechanism was embedded in the image classification model to differentiate the structure and appearance of different infections. Finally, the overall analysis report for a single CT sample was generated using the Noisy-or Bayesian function. The results show that the proposed approach could more accurately detect COVID-19 cases than without the location-attention model. Several other studies modified the CNN for COVID-19 detection. In [138], a multi-objective differential evolution-based CNN was utilised. Sedik et al. [139] implemented CNN and LSTM with data augmentation, while Ahsan et al. [140] employed MLP-CNN based model. The authors of [141] employed capsule network-based framework with transfer learning. Table 4 shows the summary of papers for COVID-19 detection using deep learning.

### 4.8. Dataset

The datasets used by the surveyed works are reported in this section. Table 5, Table 6, Table 7 and Table 8 show the summary of datasets used for tuberculosis, pneumonia, lung cancer and COVID-19 detection, respectively. This is done to provide readers with relevant information on the datasets. Note that only public datasets are included in the tables because they are available to the public, whereas private datasets are inaccessible without permission.

According to Table 5, among the twelve datasets used for tuberculosis detection works, five of them do not contain tuberculosis medical images: JSRT dataset, Indiana dataset, NIH-14 dataset, LDOCTCXR and RSNA pneumonia dataset. JSRT dataset contains lung cancer images, while the Indiana and NIH-14 datasets contain multiple different diseases. LDOCTCXR and RSNA pneumonia datasets both contain pneumonia and normal lung images. These five datasets were used for transfer learning in several studies. Models were first trained to identify abnormalities in chest X-ray, and then they were trained to identify tuberculosis. The India, Montgomery and Shenzhen datasets contain X-ray images of tuberculosis; ImageCLEF 2018 and ImageCLEF 2019 datasets contain CT images of tuberculosis; and the Belarus dataset contains both X-ray and CT images of tuberculosis. Two of the datasets contain sputum smear microscopy images of tuberculosis: the TBimages dataset and ZiehlNeelsen Sputum smear Microscopy image DataBase.

For detection works related to pneumonia, only four public datasets are available, as shown in Table 6. All four datasets contain X-ray images only. Even though the number of datasets is low, the number of images within these datasets is high. Future studies utilising these datasets should have sufficient data.

According to Table 7, among the ten datasets used for lung cancer detection works, only one contains histopathology images, which is the NCI Genomic Data Commons dataset. The NIH-14 dataset contains X-ray images, while the JSRT dataset contains a mix of X-ray and CT images. The rest of the datasets all contain CT images.

Table 8 shows that there are thirteen public datasets related to COVID-19. With the rise of the COVID-19 pandemic, multiple datasets have been made available to the public. Many of these datasets still have a rising number of images. Therefore, the number of images within the datasets might be different from the number reported in this paper. Take note that some of the images might be contained in multiple datasets. Therefore, future studies should check for duplicate images.

Table 9 summarises the works surveyed based on the taxonomy. This allows readers to quickly refer to the articles according to their interested attributes. The analysis of the distribution of works based on the identified attributes of the taxonomy is given in the following section.

## 5. Analysis of Trend, Issues and Future Directions of Lung Disease Detection Using Deep Learning

In this section, the broad analysis of the existing work is presented, which is the last contribution outlined in this paper. The analysis of the trend of each attribute identified in the foregoing section is described, whereby the aim is to show the progress of the works and the direction the researchers are heading over the last five years. The shown trend could be useful to suggest the future direction of the work in this domain. Section 5.1 presents the analysis of the trend of the articles considered. The issues and potential future work to address the identified issues are described in Section 5.2.

### 5.1. An Analysis of the Trend of Lung Disease Detection in Recent Years

This subsection presents the analysis of lung disease detection works in recent years for each attribute of the taxonomy described in the foregoing section.

#### 5.1.1. Trend Analysis of the Image Type Used

**Figure 10:**
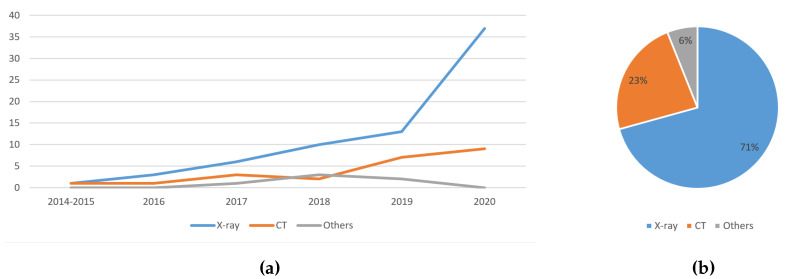
(**a**) The trend of the usage of image types in lung disease detection works in recent years; and (**b**) the distribution of the image type used in deep learning aided lung disease detection in recent years.

Figure 10a shows that the usage of X-ray images increases linearly over the years. The usage of CT images also increases over the years, with a slight dip in 2018. The sputum smear microscopy and histopathology images are combined into one as ‘Others’ due to the low number of previous work using them to detect lung diseases. The usage of other image types slowly increases until 2018, and then drops. This indicates that deep learning aided lung disease detection works are heading towards the direction of using X-ray images and CT images.

Figure 10b shows that the majority of the studies used X-ray images at 71%, while CT images followed second with 23%. Such observation could be due to the availability, accessibility and mobility of X-ray machines over the CT scanner. Due to the COVID-19 pandemic that has spread to all types of geographical locations, it is anticipated that the X-ray images will still be the dominant choice of medical images used to detect lung-related diseases over CT images. CT images may remain the second choice because they provide more detailed information than X-rays.

#### 5.1.2. Trend Analysis of the Features Used

**Figure 11:**
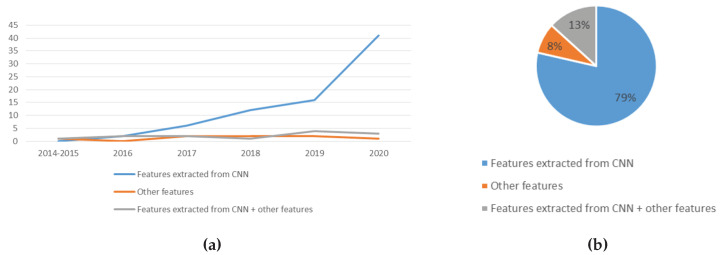
(**a**) The trend of the usage of features in lung disease detection works in recent years; and (**b**) the distribution of usage of data augmentation in deep learning aided lung disease detection in recent years.

From the perspective of features used for lung disease detection in recent years, as shown in Figure 11a, the usage of CNN extracted features is steadily increasing, while the usage of other features and the combination of CNN extracted features plus other features remain low. This is because CNN allows automated feature extraction, discarding the need for manual feature generation [40]. The usage of other features was less preferred due to the fact that most recent works showed the superiority of CNN extracted features in detecting lung diseases. Figure 11b shows the distribution of work by type of features used. CNN extracted features were used in 79% of the works. The combination of CNN extracted features plus some other features were used in 13% of the recent works, while the remaining works utilised other types of features.

#### 5.1.3. Trend Analysis of the Usage of Data Augmentation

**Figure 12:**
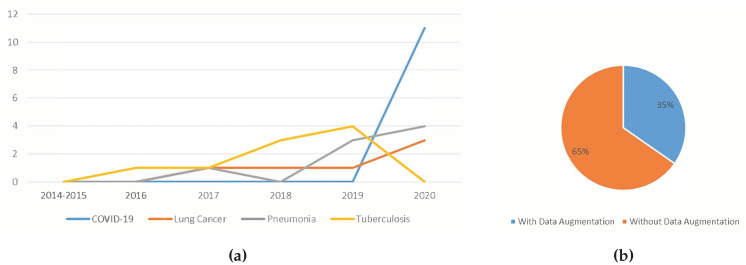
(**a**) The trend of the usage of data augmentation in lung disease detection works in recent years; and (**b**) the distribution of usage of data augmentation in deep learning aided lung disease detection in recent years.

Figure 12a shows the trend of the usage of data augmentation. Although implementing data augmentation increased the complexity of the data pre-processing, the number of works employing data augmentation increases steadily over the years. Such trend signifies that more researchers have realised how beneficial data augmentation is to train the lung disease detection models.

Figure 12b shows the distribution of data augmentation usage in deep learning aided lung disease detection. Only about one-third of the studies used data augmentation. While it is reported that data augmentation improved the classification accuracy, the majority of works did not use data augmentation. One reason for this might be that data augmentation is not that simple to implement. As mentioned in Section 4.3, the disadvantages of data augmentation include additional memory costs, transformation computing costs and training time.

#### 5.1.4. Trend Analysis of the Types of Deep Learning Algorithm Used

**Figure 13:**
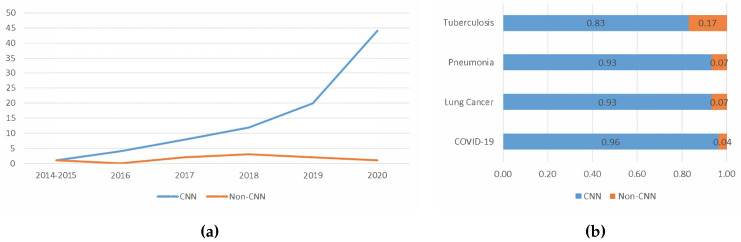
(**a**) The trend of the usage of deep learning algorithms in lung disease detection works in recent years; and (**b**) the distribution of the usage of CNN in deep learning aided lung disease detection in recent years.

Figure 13a shows the trend of the usage of deep learning algorithms in lung disease detection works in recent years. As shown in Figure 13, CNN was the most preferred deep learning algorithm for the last five years. Future works will likely follow this trend, whereby more work may prefer CNN for lung disease detection over other deep learning algorithms.

Figure 13b visualises the analysis of the usage of CNN in deep learning aided lung disease detection in recent years. The majority of the papers surveyed used CNN. This is because CNN is robust and can achieve high classification accuracy. Many of the works surveyed indicate that CNN has superior performance [74]. Other benefits of using CNN include automatic feature extraction and utilising the advantages of transfer learning, which is further analysed in the following subsection.

#### 5.1.5. Trend Analysis of the Usage Of Transfer Learning

**Figure 14:**
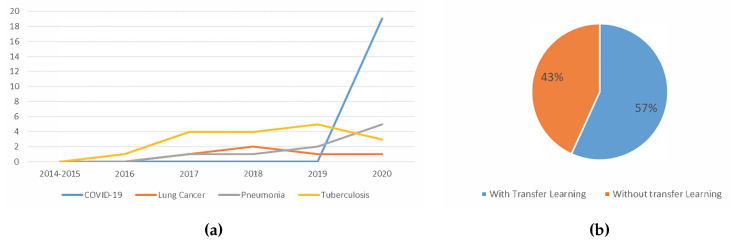
(**a**) The trend of the usage of transfer learning in lung disease detection works in recent years; and (**b**) the usage of transfer learning in lung disease detection works using CNN.

Figure 14a shows the trend of the usage of transfer learning. As time goes on, more works employed transfer learning. With transfer learning, there is no need to define a new model. Transfer learning also allows the usage features learned while training from an old task for the new task, often increasing the classification accuracy. This could be due to the model used being more generalised as it has been trained with a greater number of images.

Figure 14b shows the usage of transfer learning among the works which used CNN. According to the figure, 57% of the recent works utilised transfer learning. Even though the number of works utilising transfer learning increased over the years, as shown in Figure 14a, the percentage of works using transfer learning is just 57%. For example, in 2020, out of 44 studies that used CNN, 28 implemented transfer learning. This suggests that works in this domain are moving towards the direction of using transfer learning, but not at a high pace. Transfer learning remains a strong approach to lung disease detection, with respect to the detection performance. Hence, the distribution of work may be skewed towards transfer learning in the near future.

#### 5.1.6. Trend Analysis of the Usage Of Ensemble

**Figure 15:**
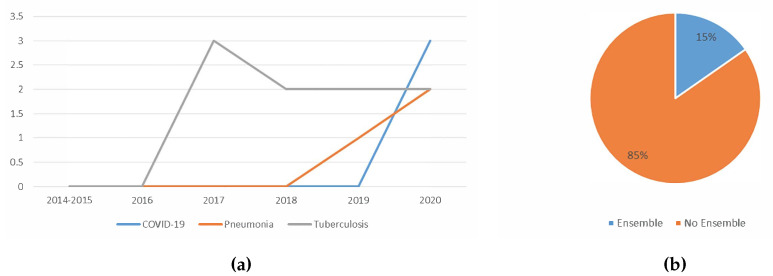
(**a**) The trend of the usage of ensemble classifier in lung disease detection works in recent years; and (**b**) the distribution of the usage of the ensemble in deep learning aided lung disease detection in recent years.

Based on Figure 15a, it seems that the ensemble was only applied on COVID-19, pneumonia and tuberculosis detection. It is observed that the usage of the ensemble is slowly growing in popularity for pneumonia and COVID-19 detection. Although less popular, the works that deployed an ensemble classifier reported better detection performance than when not using ensemble.

Figure 15b shows the distribution of the usage of the ensemble in deep learning aided lung disease detection. Only 15% of the studies used ensemble. This suggests that ensemble classifier is still less explored for lung disease detection. Only three types of ensemble techniques were found in the papers surveyed, which were majority voting, probability score averaging and stacking. The challenge to implement ensemble may be the caused of such low application. Using ensemble, the performance could only improve if the errors of the base classifiers have a low correlation. When using similar data, which may occur when the size of the datasets and the number of datasets itself are limited, the correlation of errors of the base classifiers tends to be high.

#### 5.1.7. Trend Analysis of the Type Of Lung Disease Detected using Deep Learning

**Figure 16:**
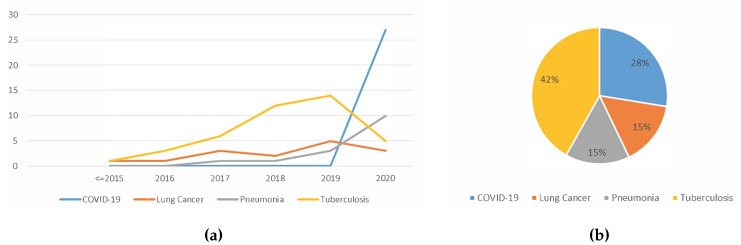
(**a**) The trend of the deep learning aided lung disease detection works in recent years; and (**b**) the distribution of the diseases detected using deep learning in recent years.

Based on the trend shown in Figure 16a, the total number of lung disease detection works using deep learning increased steadily over the years, with most work related to tuberculosis detection. As more lung disease medical image datasets become public, researchers have access to more data. Thus, more extensive studies were conducted. Towards 2020, the works on COVID-19 detection emerged while work conducted to detect other diseases decreased tremendously. This signifies that using deep learning to detect lung disease is still an active field of study. This also shows that much effort was directed towards easing the burden of detecting COVID-19 using the existing manual screening test, which is already anticipated.

Figure 16b shows the distribution of the diseases detected using deep learning in recent years. The majority of works were directed at tuberculosis detection, followed by COVID-19, lung cancer and pneumonia. The reason that works of tuberculosis are high is because the majority of tuberculosis-infected inhabitants were from resource-poor regions with poor healthcare infrastructure [61]. Therefore, tuberculosis detection using deep learning provides the opportunity to accelerate tuberculosis diagnosis among these communities. The reason that works of COVID-19 detection are second highest is because researchers all over the world are trying to reduce the burden of detecting COVID-19, and thus many works have been published, even though COVID-19 is a relatively new disease.

### 5.2. Issues and Future Direction of Lung Disease Detection Using Deep Learning

This subsection presents the remaining issues and corresponding future direction of lung disease detection using deep learning, which are the final contributions of this paper. The state-of-the-art lung disease detection field is suffering from several issues that can be found in the papers considered. Some of the proposed future works are designed to deal with the issues found. Details of the issues and potential future works are presented in Section 5.2.1 and Section 5.2.2, respectively.

#### 5.2.1. Issues

This section presents the issues of lung disease detection using deep learning found in the literature. Four main issues were identified: (i) data imbalance; (ii) handling of huge image size; (iii) limited available datasets; and (iv) high correlation of errors when using ensemble techniques.

(i) Data imbalance: When doing classification training, if the number of samples of one class is a lot higher than the other class, the resulting model would be biased. It is better to have the same number of images in each class. However, oftentimes that is not the case. For example, when performing a multiclass classification of COVID-19, pneumonia and normal lungs, the number of images for pneumonia far exceeds the number of images for COVID-19 [126].
(ii) Handling of huge image size: Most researchers reduced the original image size during training to reduce computational cost. It is extremely computationally expensive to train with the original image size, and it is also time-consuming to train a deeply complex model even with the aid of the most powerful GPU hardware.
(iii) Limited available datasets: Ideally, thousands of images of each class should be obtained for training. This is to produce a more accurate classifier. However, due to the limited number of datasets, the number of available training data is often less than ideal. This causes researchers to search for other alternatives to produce a good classifier.
(iv) High correlation of errors when using ensemble techniques: It requires a variety of errors for an ensemble of classifiers to perform the best. The base classifiers used should have a very low correlation. This, in turn, will ensure the errors of those classifiers also will be varied. In other words, it is expected that the base classifiers will complement each other to produce better classification results. Most of the studies surveyed only combine classifiers that were trained on similar features. This causes the correlation error of the base classifiers to be high.

#### 5.2.2. Potential Future Works

This section presents the possible future works that should be considered to improvise the performance of lung disease detection using deep learning.

(i) Make datasets available to the public: Some researchers used private hospital datasets. To obtain larger datasets, efforts such as de-identification of confidential patients’ information can be conducted to make the data public. With more data available, the produced classifiers would be more accurate. This is because, with more data comes more diversity. This decreases the generalisation error because the model becomes more general as it was trained on more examples. Medical data are hard to come by. Therefore, if the datasets were made public, more data would be available for researchers.
(ii) Usage of cloud computing: Performing training using cloud computing might overcome the problem of handling of huge image size. On a local mid-range computer, training with large images will be slow. A high-end computer might speed up the process a little, but it might still be infeasible. However, by training the deep learning model using cloud computing, we can use multiple GPUs at a reasonable cost. This allows higher computational cost training to be conducted faster and cheaper.
(iii) Usage of more variety of features: Most researchers use features automatically extracted by CNN. Some other features such as SIFT, GIST, Gabor, LBP and HOG were studied. However, many other features are still yet to be explored, for example quadtree and image histogram. Efforts can be directed to studying different types of features. This can address the issue of the high correlation of errors when using ensemble techniques. With more features comes more variation. When combining many variations, the results are often better [41]. Feature engineering allows the extraction of more information from present data. New information is extracted in terms of new features. These features might have a better ability to describe the variance in the training data, thus improving model accuracy.
(iv) Usage of the ensemble learning: Ensemble techniques show great potentials. Ensemble methods often improve detection accuracy. An ensemble of several features might provide better detection results. An ensemble of different deep learning techniques could also be considered because ensembles perform better if the errors of the base classifiers have a low correlation.

## 6. Limitation of the Survey

The survey presented has a limitation whereby the primary source of work considered were those indexed in the Scopus database, due to the reason described in Section 2. Exceptions were given on COVID-19 related works, as most of the articles were still at the preprint level when this survey was conducted. Concerning the publication years considered, the latest publication included were those published prior to October 2020. Therefore, the findings put forward in this survey paper did not consider contributions of works that are non-Scopus indexed and those that are published commencing October 2020 and onwards.

## 7. Conclusions

As time goes on, more works on lung disease detection using deep learning have been published. However, there was a lack of systematic survey available on the current state of research and application. This paper is thus produced to offer an extensive survey of lung disease detection using deep learning, specifically on tuberculosis, pneumonia, lung cancer and COVID-19, published from 2016 to September 2020. In total, 98 articles on this topic were considered in producing this survey.

To summarise and provide an organisation of the key concepts and focus of the existing work on lung disease detection using deep learning, a taxonomy of state-of-the-art deep learning aided lung disease detection was constructed based on the survey on the works considered. Analyses of the trend on recent works on this topic, based on the identified attributes from the taxonomy, are also presented. From the analyses of the distribution of works, the usage of both CNN and transfer learning is high. Concerning the trend of the surveyed work, all the identified attributes in the taxonomy observed, on average, a linear increase over the years, with an exception to the ensemble attribute. The remaining issues and future direction of lung disease detection using deep learning were subsequently established and described. Four issues of lung disease detection using deep learning were identified: data imbalance, handling of huge image size, limited available datasets and high correlation of errors when using ensemble techniques. Four potential works for lung disease detection using deep learning are suggested to resolve the identified issues: making datasets available to the public, usage of cloud computing, usage of more features and usage of the ensemble.

To conclude, investigating how deep learning was employed in lung disease detection is highly significant to ensure future research will concentrate on the right track, thereby improving the performance of disease detection systems. The presented taxonomy could be used by other researchers to plan their research contributions and activities. The potential future direction suggested could further improve the efficiency and increase the number of deep learning aided lung disease detection applications.

## Figures and Tables

**Table 1:** Summary of papers for tuberculosis detection using deep learning.

Authors	Deep Learning Technique	Features	Dataset
[74]	CNN with transfer learning and data augmentation	Features extracted from CNN	Montgomery
[38]	K-nearest neighbour, Simple Linear Regression and Sequential Minimal Optimisation (SMO) Classification	Area, major axis, minor axis, eccentricity, mean, kurtosis, skewness and entropy	Shenzhen
[84]	ViDi	Features extracted from CNN	Unspecified
[64]	CNN	Gabor, LBP, SIFT, PHOG and Features extracted from CNN	Private dataset
[24]	CNN	Features extracted from CNN	ImageCLEF 2018 dataset
[62]	CNN with transfer learning, with demographic information	Features extracted from CNN + demographic information	Private dataset
[79]	CNN with data augmentation, and ensemble by weighted averages of probability scores	Features extracted from CNN	Montgomery, Shenzhen, Belarus, JSRT
[70]	CNN with transfer learning and data augmentation	Features extracted from CNN	Private dataset, Montgomery, Shenzhen
[69]	CNN	Features extracted from CNN	Private datasets, Montgomery, Shenzhen
[71]	CNN with transfer learning and ensemble by simple linear probabilities averaging	Features extracted from CNN + rule-based features	Indiana, JSRT, Shenzhen
[29]	CNN	HoG features	ZiehlNeelsen Sputum smear Microscopy image DataBase
[75]	CNN and shuffle sampling	Features extracted from CNN	Private datasets
[81]	CNN with transfer learning and ensemble by averaging	CNN extracted features from edge images	Montgomery, Shenzhen
[57]	CNN with transfer learning, data augmentation and ensemble by weighted probability scores average	Features extracted from CNN	Private dataset, Montgomery, Shenzhen, Belarus
[85]	AutoEncoder-CNN	Features extracted from CNN	Private dataset
[76]	CNN with transfer learning and shuffle sampling	Features extracted from CNN	Private dataset
[65]	End-to-end CNN	Features extracted from CNN	Montgomery, Shenzhen
[88]	Optical flow model	Activity Description Vector on optical flow of video sequences	ImageCLEF 2019 dataset
[28]	CNN	Colours	TBimages dataset
[83]	Modified maximum pattern margin support vector machine (modified miSVM)	First four moments of the intensity distributions	Private datasets
[61]	CAD4TB with clinical information	Features extracted from CNN + clinical features	Private dataset
[31]	DBN	LoH + SURF features	ZiehlNeelsen Sputum smear Microscopy image DataBase
[60]	CAD4TB	Features extracted from CNN	Private dataset
[72]	CNN with transfer learning and data augmentation	Features extracted from CNN	Montgomery, Shenzhen, NIH-14 dataset
[30]	CNN	Features extracted from CNN	TBimages dataset
[63]	CNN from scratch and data augmentation	Features extracted from CNN	Montgomery, Shenzhen, Belarus
[86]	3D CNN	Features extracted from CNN + lung volume + patient attribute metadata	ImageCLEF 2019 dataset
[12]	CNN with transfer learning and ensemble by stacking	local and global feature descriptors + features extracted from CNN	Private dataset, Montgomery, Shenzhen, India
[80]	CNN with transfer learning and feature level ensemble	Features extracted from CNN	Shenzhen
[15]	CNN with transfer learning and ensemble by averaging	CNN extracted features from edge images	Montgomery, Shenzhen
[32]	CNN with transfer learning	Features extracted from CNN	ZiehlNeelsen Sputum smear Microscopy image DataBase
[66]	CNN with data augmentation	Features extracted from CNN	Shenzhen
[73]	CNN with transfer learning and data augmentation	Features extracted from CNN	NIH-14, Montgomery, Shenzhen
[19]	CNN with transfer learning, Bag of CNN Features and ensemble by a simple soft-voting scheme	Features extracted from CNN + BOW	Private dataset, Montgomery, Shenzhen
[36]	Neural network	Shape, curvature descriptor histograms, eigenvalues of Hessian matrix	Montgomery, Shenzhen
[77]	CNN with transfer learning and data augmentation	Features extracted from CNN	Montgomery, Shenzhen, NIH-14
[87]	3D CNN	Features extracted from CNN	ImageCLEF 2019 dataset
[78]	CNN and Artificial Ecosystem-based Optimisation algorithm	Features extracted from CNN	Shenzhen
[67]	CNN	Features extracted from CNN	Shenzhen
[68]	Bayesian based CNN	Features extracted from CNN	Montgomery, Shenzhen
[82]	CNN with transfer learning, and ensemble by majority voting, simple averaging, weighted averaging, and stacking	Features extracted from CNN	Montgomery, Shenzhen, LDOCTCXR, 2018 RSNA pneumonia challenge dataset, Indiana dataset

**Table 2:** Summary of papers for pneumonia detection using deep learning

Reference	Deep Learning Technique	Features	Dataset
[99]	Deep Siamese based neural network	CNN extracted features from the left half and right half of the lungs	Unspecified Kaggle dataset
[20]	CNN with transfer learning and data augmentation	Features extracted from CNN	LDOCTCXR
[55]	CNN with transfer learning, data augmentation and ensemble by majority voting.	Features extracted from CNN	LDOCTCXR
[93]	CNN with transfer learning	Features extracted from CNN	LDOCTCXR
[102]	CNN with transfer learning, data augmentation and ensemble by combining confidence scores and bounding boxes.	Features extracted from CNN	Radiological Society of North America (RSNA) pneumonia dataset
[96]	CNN with transfer learning and data augmentation	Features extracted from CNN	NIH Chest X-ray Dataset
[92]	CNN from scratch and data augmentation	Features extracted from CNN	LDOCTCXR
[95]	CNN with transfer learning	Features extracted from CNN	LDOCTCXR
[91]	CNN	Features extracted from CNN	Mooney’s Kaggle dataset
[100]	CNN and LSTM-CNN, with transfer learning and data augmentation	Features extracted from CNN	Mooney’s Kaggle dataset
[103]	CNN with probabilistic map of pneumonia	Features extracted from CNN	2018 RSNA pneumonia challenge dataset
[101]	Decision Tree, Random Forest, K-nearest neighbour, AdaBoost, Gradient Boost, XGBboost, CNN	Multiple features	Mooney’s Kaggle dataset
[98]	CNN with transfer learning, data augmentation and ensemble by weighted averaging	Features extracted from CNN	LDOCTCXR
[97]	CNN with transfer learning and data augmentation	Features extracted from CNN	Mooney’s Kaggle dataset
[94]	CNN with transfer learning	Features extracted from CNN	Private dataset

**Table 3:** Summary of papers for lung cancer detection using deep learning.

Reference	Deep Learning Technique	Features	Dataset
[13]	CNN	Features extracted from CNN	LUNA, LIDC, NLST
[113]	CNN with transfer learning	Features extracted from CNN	JSRT Dataset, NIH-14 dataset
[107]	Multi-stream multi-scale convolutional networks	Features extracted from CNN	MILD dataset DLCST dataset
[34]	CNN with transfer learning	Features extracted from CNN	NCI Genomic Data Commons
[110]	CNN with transfer learning and data augmentation	Features extracted from CNN	NSCLC-Radiomics, NSCLC-Radiomics-Genomics, RIDER Collections and several private datasets
[105]	CNN and DBN	Features extracted from CNN and DBN	LIDC-IDRI
[112]	CNN with transfer learning	Features extracted from CNN	Kaggle Data Science Bowl 2017 dataset, Lung Nodule Analysis 2016 (LUNA16) dataset
[25]	CNN	Features extracted from CNN	LIDC-IDRI
[108]	CNN	Features extracted from CNN	LIDC-IDRI
[23]	CNN with data augmentation	Features extracted from CNN	LIDC-IDRI database
[111]	CNN with transfer learning and data augmentation	Features extracted from CNN	Private dataset
[14]	Bone elimination and lung segmentation before training with CNN	Features extracted using CNN from bone eliminated lung images and segmented lung images	JSRT dataset
[114]	CNN-long short-term memory network	Features extracted from CNN	NIH-14 dataset
[109]	CNN with transfer learning and data augmentation	Features extracted from CNN	JSRT database
[106]	CNN with data augmentation	Features extracted from CNN	Cancer Imaging Archive

**Table 4:** Summary of papers for COVID-19 detection using deep learning.

Authors	Deep Learning Technique	Features	Dataset
[137]	CNN with transfer learning and location-attention classification mechanism	Features extracted from CNN	Private dataset
[125]	CNN with transfer learning and data augmentation	Features extracted from CNN	SIRM database, Cohen’s Github dataset, Chowdhury’s Kaggle dataset
[26]	RADLogics Inc., CNN with transfer learning and data augmentation	Features extracted from RADLogics Inc and CNN	Chainz Dataset, A dataset from a hospital in Wenzhou, China, Dataset from El-Camino Hospital (CA) and Lung image database consortium (LIDC)
[123]	CNN with transfer learning	Features extracted from CNN	Cohen’s Github dataset and LDOCTCXR
[21]	CNN with transfer learning and data augmentation	Features extracted from CNN	Cohen’s Github dataset and unspecified Kaggle dataset
[135]	VB-Net and modified random decision forests method	96 handcrafted image features	Dataset obtained from Tongji Hospital of Huazhong University of Science and Technology, Shanghai Public Health Clinical Center of Fudan University, and China-Japan Union Hospital of Jilin University.
[126]	CNN from scratch and data augmentation	Features extracted from CNN	COVIDx Dataset
[127]	CNN with transfer learning	Features extracted from CNN	Cohen’s Github dataset, Andrew’s Kaggle dataset, LDOCTCXR
[117]	CNN with transfer learning	Features extracted from CNN	Cohen’s Github dataset, RSNA pneumonia dataset, COVIDx
[131]	CNN with transfer learning and data augmentation	Features extracted from CNN	Sajid’s Kaggle dataset
[4]	CNN with transfer learning and data augmentation	Features extracted from CNN	Cohen’s Github dataset, Mooney’s Kaggle dataset
[118]	CNN with transfer learning	Features extracted from CNN	COVID-CT-Dataset
[128]	CNN as feature extractor and long short-term memory (LSTM) network as classifier	Features extracted from CNN	GitHub, Radiopaedia, The Cancer Imaging Archive, SIRM, Kaggle repository, NIH dataset, Mendeley dataset
[132]	CNN with transfer learning and synthetic data generation and augmentation	Features extracted from CNN	Cohen’s Github, Chowdhury’s Kaggle dataset, COVID-19 Chest X-ray Dataset, Initiative
[129]	CNN with transfer learning, data augmentation and ensemble by majority voting	Features extracted from CNN	Cohen’s Github, LDOCTCXR
[134]	CNN with transfer learning and stacking ensemble	Features extracted from CNN	Private dataset, LDOCTCXR
[130]	CNN	Features extracted from CNN	Private dataset
[138]	Multi-objective differential evolution-based CNN	Features extracted from CNN	Unspecified
[119]	CNN with transfer learning	Features extracted from CNN	Cohen’s Github
[139]	CNN and ConvLSTM with data augmentation	Features extracted from CNN	Cohen’s Github, COVID-CT-Dataset
[120]	CNN with transfer learning	Features extracted from CNN	Cohen’s Github
[133]	CNN with ensemble by weighted averaging	Features extracted from CNN	Private hospital datasets
[121]	CNN with transfer learning	Features extracted from CNN	Cohen’s Github, Mooney’s Kaggle dataset, Shenzhen and Montgomery datasets
[140]	MLP-CNN based model	Features extracted from CNN	Cohen’s Github
[122]	CNN with transfer learning	Features extracted from CNN	Cohen’s Github, unspecified Kaggle dataset
[141]	Capsule Network-based framework with transfer learning	Features extracted from CNN	Cohen’s Github, Mooney’s Kaggle dataset

**Table 5:** Summary of datasets used for tuberculosis detection.

Name	Disease	Image Type	Reference	Number of Images	Link
Belarus dataset	Tuberculosis	X-ray and CT	[142]	1299	http://tuberculosis.by
ImageCLEF 2018 dataset	Tuberculosis	CT		2287	https://www.imageclef.org/2018/tuberculosis
ImageCLEF 2019 dataset	Tuberculosis	CT	[143]	335	https://www.imageclef.org/2019/medical/tuberculosis
India	Tuberculosis	X-ray	[39]	78 tuberculosis and 78 normal	https://sourceforge.net/projects/tbxpredict/
Indiana Dataset	Multiple diseases with annotations	X-ray	[144]	7284	https://openi.nlm.nih.gov
JSRT dataset	Lung nodules and normal	X-ray and CT	[145]	154 nodule and 93 non-nodule	http://db.jsrt.or.jp/eng.php
Montgomery and Shenzhen datasets	Tuberculosis and normal	X-ray	[146]	394 tuberculosis and 384 normal	https://lhncbc.nlm.nih.gov/publication/pub9931
NIH-14 dataset	Pneumonia and 13 other diseases	X-ray	[147]	112120	https://www.kaggle.com/nih-chest-xrays/data
TBimages dataset	Tuberculosis	Sputum smear microscopy image	[148]	1320	http://www.tbimages.ufam.edu.br/
ZiehlNeelsen Sputum smear Microscopy image DataBase	Tuberculosis	Sputum smear microscopy image	[27]	620 tuberculosis and 622 normal	http://14.139.240.55/znsm/
Large Dataset of Labeled Optical Coherence Tomography (OCT) and Chest X-Ray Images (LDOCTCXR)	Pneumonia and normal	X-ray	[93]	3883 pneumonia and 1349 normal	https://data.mendeley.com/datasets/rscbjbr9sj/3
Radiological Society of North America (RSNA) pneumonia dataset	Pneumonia and normal	X-ray		5528	https://www.kaggle.com/c/rsna-pneumonia-detection-challenge/data

**Table 6:** Summary of datasets used for pneumonia detection.

Name	Disease	Image Type	Reference	Number of Images	Link
LDOCTCXR	X-ray	[93]	3883 pneumonia and 1349 normal	https://data.mendeley.com/datasets/rscbjbr9sj/3	
NIH Chest X-ray Dataset	Pneumonia and 13 other diseases	X-ray	[147]	112,120	https://www.kaggle.com/nih-chest-xrays/data
Radiological Society of North America (RSNA) pneumonia dataset	Pneumonia and normal	X-ray		5528	https://www.kaggle.com/c/rsna-pneumonia-detection-challenge/data
Mooney’s Kaggle dataset	Pneumonia and normal	X-ray		5863	https://www.kaggle.com/paultimothymooney/chest-xray-pneumonia

**Table 7:** Summary of datasets used for lung cancer detection.

Name	Disease	Image Type	Reference	Number of Images	Link
JSRT dataset	Lung nodules and normal lungs	X-ray and CT	[145]	154 nodule and 93 non-nodule	http://db.jsrt.or.jp/eng.php
Kaggle Data Science Bowl 2017 dataset	Lung Cancer	CT scans		601	https://www.kaggle.com/c/data-science-bowl-2017/overview
LIDC-IDRI	Lung Cancer	CT	[149]	1018	https://wiki.cancerimagingarchive.net/display/Public/LIDC-IDRI
Lung Nodule Analysis 2016 (LUNA16) dataset	Location and size of lung nodules	CT scans	[8]	888	https://luna16.grand-challenge.org/download/
NCI Genomic Data Commons	Lung Cancer	histopa- thology images	[150]	More than 575,000	https://portal.gdc.cancer.gov/
NIH-14 dataset	14 lung diseases	X-ray	[147]	112,120	https://www.kaggle.com/nih-chest-xrays/data
NLST	Lung Cancer	CT		Approximately 200,000	https://biometry.nci.nih.gov/cdas/learn/nlst/images/
NSCLC-Radiomics	Lung Cancer	CT		422	https://wiki.cancerimagingarchive.net/display/Public/NSCLC-Radiomics
NSCLC- Radiomics -Genomics	Lung Cancer	CT		89	https://wiki.cancerimagingarchive.net/display/Public/NSCLC-Radiomics-Genomics
RIDER Collections	Lung Cancer	CT		Approximately 280,000	https://wiki.cancerimagingarchive.net/display/Public/RIDER+Collections

**Table 8:** Summary of datasets used for COVID-19 detection.

Name	Disease	Image Type	Reference	Number of Images	Link
Andrew’s Kaggle dataset	COVID-19	X-ray and CT		79	https://www.kaggle.com/andrewmvd/convid19-x-rays
Chainz Dataset	COVID-19 and normal	CT		50 COVID-19, 51 normal	www.ChainZ.cn
Chowdhury’s Kaggle dataset	COVID-19, normal and pneumonia	X-ray	[125]	219 COVID-19, 1341 normal and 1345 pneumonia	https://www.kaggle.com/tawsifurrahman/covid19-radiography-database
Cohen’s Github dataset	COVID-19	X-ray and CT	[151]	123	https://github.com/ieee8023/covid-chestxray-dataset
COVIDx Dataset	COVID-19, normal and pneumonia	X-ray	[126]	573 COVID-19, 8066 normal and 5559 pneumonia	https://github.com/lindawangg/COVID-Net/blob/master/docs/COVIDx.md
Italian Society Of Medical And Interventional Radiology (SIRM) COVID-19 Database	COVID-19	X-ray and CT		68	https://www.sirm.org/category/senza-categoria/covid-19/
LDOCTCXR	Pneumonia and normal	X-ray	[93]	3883 pneumonia and 1349 normal	https://data.mendeley.com/datasets/rscbjbr9sj/3
Lung image database consortium (LIDC)	Lung Cancer	CT	[149]	1018	https://wiki.cancerimagingarchive.net/display/Public/LIDC-IDRI
Sajid’s Kaggle dataset	COVID-19 and normal	X-ray		28 normal, 70 COVID-19	https://www.kaggle.com/nabeelsajid917/covid-19-x-ray-10000-images
Mooney’s Kaggle dataset	Pneumonia and normal	X-ray		5863	https://www.kaggle.com/paultimothymooney/chest-xray-pneumonia
COVID-CT Dataset	COVID-19 and normal	CT		349 COVID-19 and 463 non-COVID-19	https://github.com/UCSD-AI4H/COVID-CT
Mendeley Augmented COVID-19 X-ray Images Dataset	COVID-19 and normal	X-ray		912	https://data.mendeley.com/datasets/2fxz4px6d8/4
COVID-19 Chest X-Ray Dataset Initiative	COVID-19	X-ray		55	https://github.com/agchung/Figure1-COVID-chestxray-dataset

**Table 9:** Summary of the works surveyed based on the taxonomy.

Attributes	Subattributes	References
Image types	X-Ray	[4,12,14,15,19,20,21,24,36,38,55,57,60,61,62,63,64,65,66,67,68,69,70,71,72,73,74,75,76,77,78,79,80,81,82,83,84,85,91,92,93,94,95,96,97,98,99,100,101,102,103,109,113,114,117,119,120,121,122,123,124,125,126,127,128,129,131,132,134,139,140,141]
	CT Scans	[13,23,25,26,86,87,88,105,106,107,108,110,111,112,118,130,133,135,137,138,139]
	Sputum Smear Microscopy Images	[28,29,30,31,32]
	Histopathology images	[34]
Features	Extracted from CNN	[4,12,13,14,15,19,20,21,23,24,25,26,30,32,34,55,57,60,61,62,63,64,65,66,67,68,69,70,71,72,73,74,75,76,77,78,79,80,81,82,84,85,86,87,91,92,93,94,95,96,97,98,99,100,101,102,103,105,106,107,108,109,110,111,112,113,114,117,118,119,120,121,122,123,124,125,126,127,128,129,130,131,132,133,134,137,138,139,140,141]
	Others	[12,15,26,28,29,31,36,38,61,62,64,71,81,83,86,88,105,135]
Data augmentation	Yes	[4,20,21,23,26,55,57,63,66,70,73,74,77,79,92,96,97,98,100,102,106,109,110,111,114,122,125,126,128,129,131,132,139]
Types of deep learning algorithm	CNN	[4,12,13,14,15,19,20,21,23,24,25,26,28,29,30,32,34,55,57,60,61,62,63,64,65,66,67,68,69,72,74,76,77,78,79,80,81,82,84,85,86,91,92,93,94,95,96,97,98,99,100,101,102,103,105,106,107,108,109,110,111,112,113,114,117,118,119,120,121,122,123,124,125,126,127,128,129,130,131,132,133,134,137,138,139,140,141]
	Non-CNN	[19,26,31,36,38,83,88,105,135]
Transfer learning	Fixed feature extractor	[12,15,19,21,62,70,76,78,80,81,93,94,96,100,102,117,127,128,137]
	Fine-tuning CNN	[4,20,26,32,34,55,57,71,72,73,74,76,77,79,82,95,97,98,102,109,110,111,112,113,118,119,120,121,122,123,124,125,129,131,132,134,141]
Ensemble	Majority voting	[19,55,82,129]
	Probability score averaging	[15,57,71,79,81,82,98,102,133]
	Stacking	[12,82,134]
	Other	[80]
Disease types	Tuberculosis	[12,15,19,24,28,29,30,31,32,36,38,57,60,61,62,63,64,65,66,67,68,69,70,71,72,73,74,75,76,77,78,79,80,81,82,83,84,85,86,87,88]
	Pneumonia	[20,55,91,92,93,94,95,96,97,98,99,100,101,102,103]
	Lung cancer	[13,14,23,25,34,105,106,107,108,109,110,111,112,113,114]
	COVID-19	[4,21,26,117,118,119,120,121,122,123,124,125,126,127,128,129,130,131,132,133,134,135,137,138,139,140,141]
